# Disturbed circadian rhythm in ICU patients as indicated by melatonin levels: a prospective pilot study

**DOI:** 10.1186/cc14629

**Published:** 2015-03-16

**Authors:** K Kiss, I Földesi, L Kemény, V Csernus, Z Molnár, J Singer

**Affiliations:** 1 University of Szeged, Hungary; 2 University of Pécs, Hungary; 3 Hungarian Society for Clinical Biostatistics, Hungary

## Introduction

The aim of this prospective pilot study was to test how melatonin levels follow the circadian cycle in ICU patients. There is strong evidence that changes of circadian rhythm are reflected in melatonin levels with peak levels at dawn and low levels during daytime [1]. The ICU stay is accompanied by disturbed circadian rhythm [2], which could potentially be the result of artificial lighting conditions.

## Methods

Melatonin levels were determined in eight medical ICU patients on mechanical ventilation, without brain injury or infection. Arterial blood samples were taken on the day of admission at 18:00 (TE0) and 03:00 in the morning (TM0), then at the same time points 48 hours later (TE1, TM1). Blood samples were centrifuged at 3,000 rpm for 8 minutes, and then serum samples were stored at -80°C. Measurements were performed using a US-CEA908Ge melatonin ELISA kit. For statistical analysis, a binary (yes/no) variable was created from the pairs for each day, assigning 'Yes' if the TE values were greater than TM (melatonin peak reversion). The proportion of reversals and their 95% confidence intervals were estimated using a GEE model for repeated binary data, assuming a binomial distribution and log link, and accounting for subject as a repetition factor. All calculations were done in SAS 9.4.

## Results

Melatonin levels were not normally distributed and were ranging between 9.2 and 23.7 pg/ml at T0 and 11.3 and 17.9 pg/ ml at T1. The median differences of the morning and evening serum melatonin levels were: TM0 - TE0 = -2.8 (-3.8 - (-0.2)); TM1 - TE1 = -1.2 (-2.5 - (-0.4)) presented as median (IQR). The proportion of subjects with peak reversals was 92.9% (80.8 to 100.0%).

## Conclusion

Our preliminary data suggest that circadian rhythm disturbances may occur in critically ill patients within 48 hours after admission, and can be detected by inversion of melatonin peaks. Despite the limitations of this study, it may justify the need for larger observational and randomized trials on the effect of light on melatonin levels and on outcomes in ICU patients.
